# Comparison of Different Lactobacilli Regarding Substrate Utilization and Their Tolerance Towards Lignocellulose Degradation Products

**DOI:** 10.1007/s00284-020-02131-y

**Published:** 2020-07-29

**Authors:** Angela Gubelt, Lisa Blaschke, Thomas Hahn, Steffen Rupp, Thomas Hirth, Susanne Zibek

**Affiliations:** 1 Institute of Interfacial Process Engineering and Plasma Technology, University Stuttgart, Nobelstraße 12, 70569 Stuttgart, Germany; 2 Present Address: Institute for Bio- and Geosciences: Plant Sciences, Forschungszentrum Jülich, Jülich, Germany; 3 Present Address: Sartorius Stedim Cellca GmbH, Ulm, Germany; 4 Industrial Biotechnology, Fraunhofer Institute of Interfacial and Bioprocess Engineering, Stuttgart, Germany; 5 Present Address: Karlsruhe Institute of Technology (KIT), Karlsruhe, Germany

## Abstract

Fermentative lactic acid production is currently impeded by low pH tolerance of the production organisms, the successive substrate consumption of the strains and/or the requirement to apply purified substrate streams. We identified *Lactobacillus brevis* IGB 1.29 in compost, which is capable of producing lactic acid at low pH values from lignocellulose hydrolysates, simultaneously consuming glucose and xylose. In this study, we compared *Lactobacillus brevis* IGB 1.29 with the reference strains *Lactobacillus brevis* ATCC 367, *Lactobacillus plantarum* NCIMB 8826 and *Lactococcus lactis* JCM 7638 with regard to the consumption of C5- and C6-sugars. Simultaneous conversion of C5- and C6-monosaccharides was confirmed for *L. brevis* IGB 1.29 with consumption rates of 1.6 g/(L h) for glucose and 1.0 g/(L h) for xylose. Consumption rates were lower for *L. brevis* ATCC 367 with 0.6 g/(L h) for glucose and 0.2 g/(L h) for xylose. Further trials were carried out to determine the sensitivity towards common toxic degradation products in lignocellulose hydrolysates: acetate, hydroxymethylfurfural, furfural, formate, levulinic acid and phenolic compounds from hemicellulose fraction. *L. lactis* was the least tolerant strain towards the inhibitors, whereas *L. brevis* IGB 1.29 showed the highest tolerance. *L. brevis* IGB 1.29 exhibited only 10% growth reduction at concentrations of 26.0 g/L acetate, 1.2 g/L furfural, 5.0 g/L formate, 6.6 g/L hydroxymethylfurfural, 9.2 g/L levulinic acid or 2.2 g/L phenolic compounds. This study describes a new strain *L. brevis* IGB 1.29, that enables efficient lactic acid production with a lignocellulose-derived C5- and C6-sugar fraction.

## Introduction

Lactic acid is a compound with a broad and promising field of application. On the one hand, it can be used as building block for polymerization to polylactic acid (PLA). On the other hand, it can be applied as platform chemical for many further products, for example acrylic acid, propylene glycol and glycerol [1–3]. Especially the increasing demand for ethyl lactate and PLA lead to a higher production of lactic acid [4]. Currently, lactic acid is produced by chemical as well as by fermentative processes. The most widely used chemical process comprises the reaction of hydrogen cyanide with acetaldehyde to lactonitrile. A succeeding addition of mineral acids leads to lactic acid which is purified by esterification and distillation followed by hydrolysis [5]. Since the chemical synthesis is based on fossil resources and includes highly toxic as well as corrosive agents, a robust and cheap biotechnological manufacture process via fermentation is highly demanded. The fermentative production is based on the microbial conversion of carbohydrates to lactic acid. Lactic acid production was reported with a variety of wild-type strains and GMO-strains, such as *Escherichia coli*, *Corynebacterium glutamicum* and several *Bacilli* [6]. However, the overwhelming majority of the research work focused on lactic acid-producing bacteria commonly belonging to the order *Lactobacillales.* Prominent examples are *Pediococcus*, *Enterococcus*, *Leuconostoc*, *Streptococcus*, *Lactobacillus* and *Lactococcus* [7]. In the past, most promising results were obtained for *Lactobacillus* species. The selection of strains for lactic acid production largely depends on the substrate. In general, starch- or saccharose-containing plants are used as substrate for the fermentative production. However, these substrates are traditionally used in food and feed industry. To avoid competition with food and feed industry, other sources of substrates have to be identified. Since lignocellulose is the most abundant feedstock on earth [8], it represents a promising alternative. Unfortunately, the release of fermentable carbohydrates from lignocellulose is much more difficult than the saccharification of starch or other storage carbohydrates [9]. The reason for this is the rigid structure and complex composition of lignocellulose which consists of lignin, cellulose and hemicellulose. Microfibrils of cellulose are embedded in a matrix of lignin and hemicellulose, causing the need for biomass pretreatment prior to the enzymatic hydrolysis to generate fermentable mono- and disaccharides [10]. Pretreatment procedures include different chemical, physical, physico-chemical and biological methods [11]. Drawback of almost all treatments is the formation of potentially toxic or inhibiting compounds such as acetate, hydroxymethylfurfural (HMF), furfural, formate, levulinic acid and phenolic compounds due to the harsh reaction conditions [12–14]. Depending on the pretreatment method, the concentrations of degradation products differ. A well-known pretreatment method is the organosolv process that results in the solubilization and thus separation of hemicellulose together with lignin in a liquid fraction and cellulose as remaining solid fraction [15]. Lignin can be removed from the hemicellulose fraction by precipitation, enzyme treatment and/or adsorber application [16]. However, each purification step increases the expenses and thus the price of the product. The utilisation of a crude hemicellulose fraction as substrate for lactic acid production, including potentially toxic degradation products would be more economically. Therefore, robust strains for lactic acid production from lignocellulose-hydrolysates are required. Tu et al. [17] reported on a *Lactobacillus plantarum* isolate, which tolerated 8 g/L furfural and 6 g/L HMF at pH 5.0. van der Pol et al. [18] investigated the effect of different inhibitors on growth of *Lactobacillus casei* DSM 20011, *Lactobacillus delbrueckii* DSM20073, *Lactococcus lactis* DSM 20481, *Bacillus coagulans* DSM 2314 and *Bacillus smithii* DSM 4216. They showed 39% growth for *L. lactis* at 2.5 g/L HMF and a reduction to 4% at 5 g/L compared to the cultivation without inhibitors; a reduction of the growth performance to 37% was detected at 2.5 g/L furfural decreasing to 7% at 5 g/L. In contrast to that, low acetic acid concentrations fostered growth (122% at 5 g/L).

In our work we present a recently isolated strain, *Lactobacillus brevis* IGB 1.29 and compared its robustness towards several lignocellulose-derived inhibitors with other lactic acid-producing strains. *Lactococcus lactis* IO-1 JCM 7638 and *Lactobacillus plantarum* NCIMB 8826 were selected as representatives of the facultative hetero-fermentative strains, *Lactobacillus brevis* ATCC 367 as a commercial available strain within the same *Lactobacillus* species*.* To the best of our knowledge, such a comprehensive investigation resulting in dose-response curves for all of these strains has not been performed so far. Determination of specific inhibitory concentrations for different compounds enables the prospective pre-assessment of complex substrates for lactic acid production utilising the already stated bacteria.

On the other hand, we investigated growth performance at different pH values (pH 4 and 6) since lowering the pH does increase the amount of protonated lactic acid. The opportunity to produce lactic acid at lower pH values requires a lower amount of base for neutralization and facilitates downstream processing. However, growth at low pH also affects microbial growth due to potential toxic effects of protonated acids [19–25]. The low pH, therefore, may also affect the toxic effects of lignocellulose degradation products on the microorganisms.

After the pretreatment of lignocellulose with the organsolv process, solid and liquid fraction can be saccharified resulting in a mixture of C5- and C6-sugars, mainly glucose and xylose. Besides glucose, *L. brevis* and *L. lactis* IO-1 were able to utilize xylose as substrate [26, 27], whereas *L. plantarum* is not able to use xylose unless genetically modified [27].

A mixture of glucose and xylose often leads to diauxic cell growth because of carbon catabolite repression, which was already confirmed for *L. lactis* IO-1 [28]. Interestingly, *L. brevis* utilises glucose and xylose as co-metabolites in parallel [27, 29]. The newly isolated *L. brevis* IGB 1.29 was also analyzed with regard to co-metabolism of glucose and xylose.

## Materials and Methods

### Chemicals

All compounds applied were of technical grade. pH adjustment was performed with 2 M NaOH or 2 M HCl either. As not stated otherwise, deionized water was used.

### Pretreatment of Lignocellulose

Wheat straw was pretreated with 1 kg acetone per kg solid for 1 h at 80 °C. After separation of acetone, the solid fraction was suspended in 50% ethanol. The resulting approach was incubated for 2 h at 220 °C and 40 bar (organosolv process). The liquid extract was separated and concentrated via vacuum distillation (20-fold), resulting as hemicellulose (HC) fraction with different concentrations of the degradation products (phenolic compounds 5.1 g/L, acetate 2.2 g/L, furfural 0.01 g/L, formate 1.9 g/L, HMF 0.1 g/L, levulinic acid 0.2 g/L). The HC-fraction was applied to generate the dose-response-curves related to the phenolic compound concentration. The content of the phenolic compounds was measured with an adapted Folin–Ciocalteau assay [30] applying vanillin as a standard. The work was performed at Fraunhofer ICT (Pfinztal, Germany).

### Microorganism

*Lactococcus lactis* IO-1 JCM 7638, *Lactobacillus brevis* ATCC 367 and *Lactobacillus plantarum* NCIMB 8826 were obtained from official data collections. *Lactobacillus brevis* IGB 1.29 was isolated by the authors from garden compost.

### Isolation and Determination of *L. brevis* IGB 1.29

To isolate lactic acid-producing acid-tolerant bacteria, the enrichment was performed in low pH-medium. 100 mL defined enrichment medium (5 g/L yeast extract, 10 g/L trypton, 15 g/L glucose, 9.6 g/L citrate, pH 4) was inoculated with a garden compost sample suspended and homogenized in 1 mL tap water. The cultivation was performed in 500 mL shake flasks with baffles at 30 °C and 120 rpm (Multitron incubation shaker, Infors HT, Switzerland). If glucose in the broth was completely consumed during the screening, additional glucose was added. Optical density at a wavelength of 625 nm (OD_625_), glucose concentration, lactic acid concentration and pH value were determined and observed during microbial growth. In case of lactic acid production, which was analysed by chromatographic determination, the mixed culture was subjected to serial dilution (10^−5^, 10^−6^, 10^−7^ and 10^−8^) and streaked out on agar plates (2% agar) with the enrichment medium already used for growth in liquid suspension. The isolated single colonies were transferred to a test tube with 5 mL enrichment medium and incubated for 48 h at 120 rpm and 30 °C. Colonies were investigated concerning the formation of lactic acid: Lactic acid formers were streaked out again, repeating three times the mentioned procedure to achieve a pure culture, which was identified as *L. brevis* IGB 1.29 via ribotyping by the DSMZ (Deutsche Sammlung von Mikroorganismen und Zellkulturen, Braunschweig).

### Cultivation of Microorganisms

Seed cultures of the different strains were prepared by picking a colony from an agar plate and putting it into a test tube with 5 mL MRS medium [31]. *Lactobacilli* were incubated at 30 °C (*L. lactis* at 37 °C) and stirred at 110 rpm until the exponential phase was reached. Standard cultivations were in general performed with MRS medium at initial pH 6, 30 °C and 110 rpm (Multitron incubation shaker, Infors HT, Switzerland). Cultivations for the determination of the pH tolerance were performed at initial pH values of 4 and 6.

### Determination of Glucose/Xylose-Metabolism

Investigations concerning the consumption of monosaccharides were performed at a total carbohydrate concentration between 18 and 25 g/L. However, for *L. lactis*, a total carbohydrate concentration of 3 g/L was used since growth is greatly impeded at higher carbohydrate concentrations, probably due to high sensitivity to lower pH values based on acetic acid product formation during cultivation. To confirm the ability for simultaneous consumption, a mixture of glucose and xylose was added in a total concentration of 20 g/L. Cultures were inoculated with the seed cultures resulting in an OD_625_ of 0.04. The measurement of OD_625_ (cell growth) and monosaccharide concentration was performed with incubation until OD_625_ or monosaccharide concentrations converged to a constant value.

### Cultivation in Presence of Lignocellulose Degradation Products

The tolerance of the different strains concerning potential lignocellulose degradation products HMF, furfural, formate, levulinic acid and acetate was determined. Dose-response curves were used to identify certain threshold values for the growth with increasing concentrations of the inhibitors. Thus, different concentrations of the model substances were used to describe a full inhibition curve for each individual strain; the substances were applied in MRS medium: Acetate 0–91 g/L, furfural 0–37 g/L, formate 0–56 g/L, HMF 0–52 g/L, levulinic acid 0–133 g/L. Additionally, the influence of phenolic compounds contained in the HC fraction derived from the pretreatment of wheat straw was investigated in order to assess the sensitivity of the microorganisms towards this compound class. The HC fraction contained phenolics as major inhibitory compound and was applied in a concentration range from 0 to 6 g/L. To compare the toxicity of lignocellulose degradation products at different pH values, all experiments with different substance concentration were performed at pH 4 and pH 6. To evaluate the results, inhibitory concentration (IC) values were determined: IC_10_, IC_50_ and IC_90_, which describe the concentration of a model substance that leads to 10%, 50% and 90% inhibition of cell growth. Microorganisms were cultivated in deep well plates (2 mL volume). Main cultures were inoculated with seed cultures resulting in an OD_625_ of 0.04. All experiments were performed in duplicates. Concentration analysis of the investigated compounds was performed at the beginning and in the stationary phase.

### Analysis

Measurement of OD_625_ was performed with the photometer Genesys 10 UV (Thermo Fisher Scientific, USA). pH was measured applying pH indicator strips pH 0–10 (Merck, Germany). Glucose concentration was determined using Diabur Test 5000 (Roche Diagnostics, Switzerland) or HPLC. HPLC analysis of xylose, acetate, ethanol, lactate, HMF, furfural, levulinic acid and formate was performed with the column Aminex HPX-87 H (Bio-Rad, USA) and quantified using a refractive index detector 8120 (Bischoff, Germany). The volumetric flow rate of the mobile phase (5 mmol/L sulfuric acid) was 0.4 or 0.6 mL/min at 65 °C for different detection times up to 45 min depending on the product to be determined.

## Results and Discussion

### Co-metabolism of Glucose and Xylose

The general ability of the microorganisms listed in Materials and Methods to utilise glucose or xylose was preliminary investigated (data not shown). Except for *L. plantarum*, all microorganisms analysed were able to utilise both sugars. *L. plantarum* in general is not able to consume xylose, however, Okano et al. [32] reported genetic engineering works, enabling a potential xylose and thus hemicellulose utilisation by this strain. Nevertheless, we decided to subject *L. plantarum* to co-metabolism studies as negative control.

**Fig. 1:**
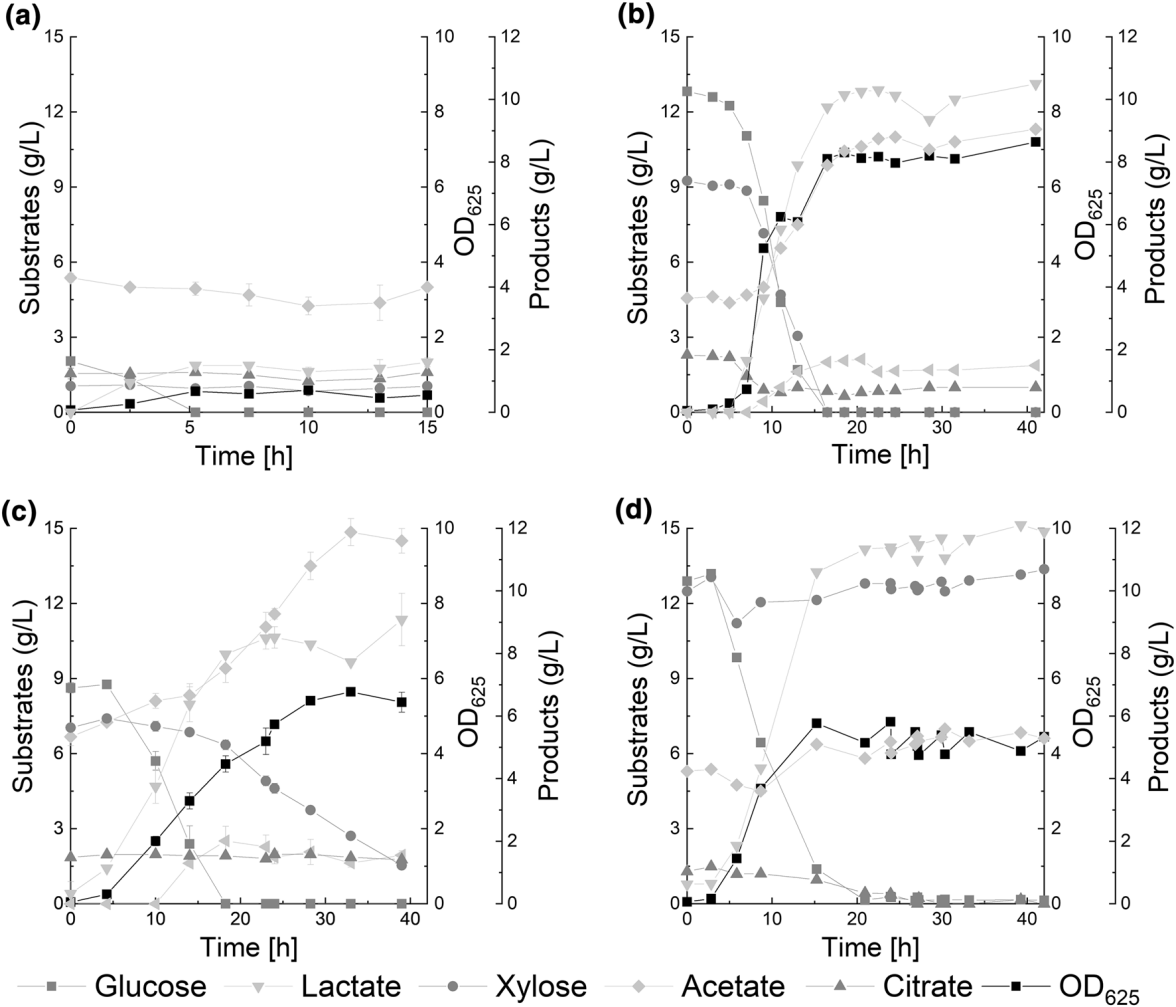
Cometabolism of glucose and xylose of **a** *L. lactis* IO-1 (JCM 7638), **b** *L. brevis* IGB 1.29, **c** *L. brevis* ATCC 367, *L. plantarum* NCIMB 8826 and substrate (dark grey) and product (light grey) concentrations were determined by HPLC analysis

The co-metabolism of glucose and xylose was analysed by supplementing both monosaccharides. Growth of the investigated lactic acid bacteria as well as the utilisation of the sugar substrates and product formation are shown in Fig. 1.

Both *L. brevis* strains were able to consume glucose and xylose simultaneously. However, a difference between the two strains was found in the maximum sugar consumption rates (see Table 1): *L. brevis* IGB 1.29 demonstrated a consumption rate of 1.0 g/(L h) for xylose which was about two thirds of the glucose consumption rate. *L. brevis* ATCC 367 consumed xylose at a maximum rate of 0.2 g/(L h) representing only about one third of the glucose consumption rate. *L. plantarum* and *L. lactis* were not able to metabolize xylose in presence of glucose even after glucose depletion. The *L. lactis* strain used had already demonstrated growth on xylose and glucose as single substrate in our hands. Therefore, the co-metabolism of both monosaccharides was investigated in more detail. First, the microorganism digested the available glucose. Due to CCR, which had already been reported for *L. lactis* IO-1 [33, 34], this strain did not co-metabolise glucose and xylose. Its inability to consume xylose after glucose depletion was unexpected and probably due to a decrease in pH to pH 5.5 and the presence of free lactic acid.

**Table 1:** Substrate consumption and product formation by *L. brevis* IGB 1.29., *L. brevis* ATCC 367, *L. plantarum* NCIMB 8826 and *L. lactis* IO-1 JCM 7638 during cometabolism of glucose and xylose

	*L. brevis* IGB 1.29 (32 h)	*L. brevis* ATCC 367 (39 h)	*L. plantarum* (39 h)	*L. lactis* (15 h)
*Glucose consumption*				
(g/L)	12.8 ± 0	8.6 ± 0.2	12.9 ± 0	2.1 ± 0.07
(%)	100	100	100	100
*Xylose consumption*				
(g/L)	9.25 ± 0	7.9 ± 0.14	0	0
(%)	100	68.4	0	0
*Y*_*Lactate/sugar*_				
(g/g)	0.5	0.5	0.9	0.5
*Y*_*Lactate*_*/OD*_*625*_				
[g/OD_625_]	1.5	1.7	3	2
*r(glucose)*				
[g/(L h)]	1.6	0.6	0.7	0.4
*r(xylose)*				
[g/(L h)]	1.0	0.2	0	0

The isolated lactic acid bacterium *L. brevis* IGB 1.29 is a heterofermentative lactic acid bacteria. It produced 10 g/L of the main product lactate and in addition 5 g/L acetate and 1.4 g/L ethanol. The same product spectrum was observed for *L. brevis* ATCC 367 with concentrations of 9.1 g/L lactate, 6.3 g/L acetate and 1.6 g/L ethanol. The yield of lactate with regard to the consumed monosaccharides was 0.5 g/g for both microorganisms, and related to OD_625_ the yield was 1.5 g/OD_625_ and 1.7 g/OD_625_, respectively. Because of the homofermentative metabolism of *L. plantarum* and *L. lactis*, they produce lactate as only product and thus yielded the highest coefficients of 0.9 g/g and 0.5 g/g with regard to the substrates and 3 g/OD_625_ and 2 g/OD_625_ with regard to the OD, respectively.

The results confirmed the findings of other authors describing the ability of all *L. brevis* species to co-ferment glucose and xylose simultaneously [27, 29, 35–37]. On the other hand, the observed sugar consumption rates were strain-specific characteristics: *L. brevis* IGB 1.29 consuming glucose 2.7 times faster and xylose 5 times faster than *L. brevis* ATCC 367. Consumption rates of related *L. brevis* strains were reported to be between those for the strains in this work, with maximum rates of 0.5 g/(L h) for glucose and 0.47 g/(L h) for xylose [35]. This qualifies *L. brevis* IGB 1.29 as a potential candidate for utilisation of lignocellulose hydrolysates as substrate. However, the simultaneous consumption of glucose and xylose is known for numerous *L. brevis* strains [35, 38]. The authors concluded that a relaxation of xylose-depending CCR might be responsible for this. *L. brevis* uses a specific H^+^-symporter which exploits the proton motive force for sugar transport [39–41]. In presence of glucose, the transport mechanism of a secondary monosaccharide switches from the H^+^-symporter to facilitated diffusion. As a result, the secondary sugar transport follows the concentration gradient so that, as long as the concentration is higher in the culture medium than in the cytosol, a flux of the sugar into the cytosol is maintained. The remaining repressing mechanism is apparently not strong enough to inhibit xylose metabolism. Due to this favourable trait, *L. brevis* is a promising candidate for lactate production from lignocellulose hydrolysates. Other microorganisms, like *Bacillus subtilis*, *Clostridium acetobutylicum*, *Kluyveromyces marxianus* or *S. cerevisiae* (genetically modified) were reported to be negatively affected by CCR during cultivation in presence of both, xylose and glucose [42–45].

*L. plantarum* did not metabolize xylose which had already been shown by several authors [46–49]. Attempts to genetically modify this species resulted in a strain which was able to co-metabolise glucose and xylose [32]. However, it was described that the specific consumption rate for xylose was less than 50% of that of glucose.

### Influence of Lignocellulose Degradation Products

**Fig. 2:**
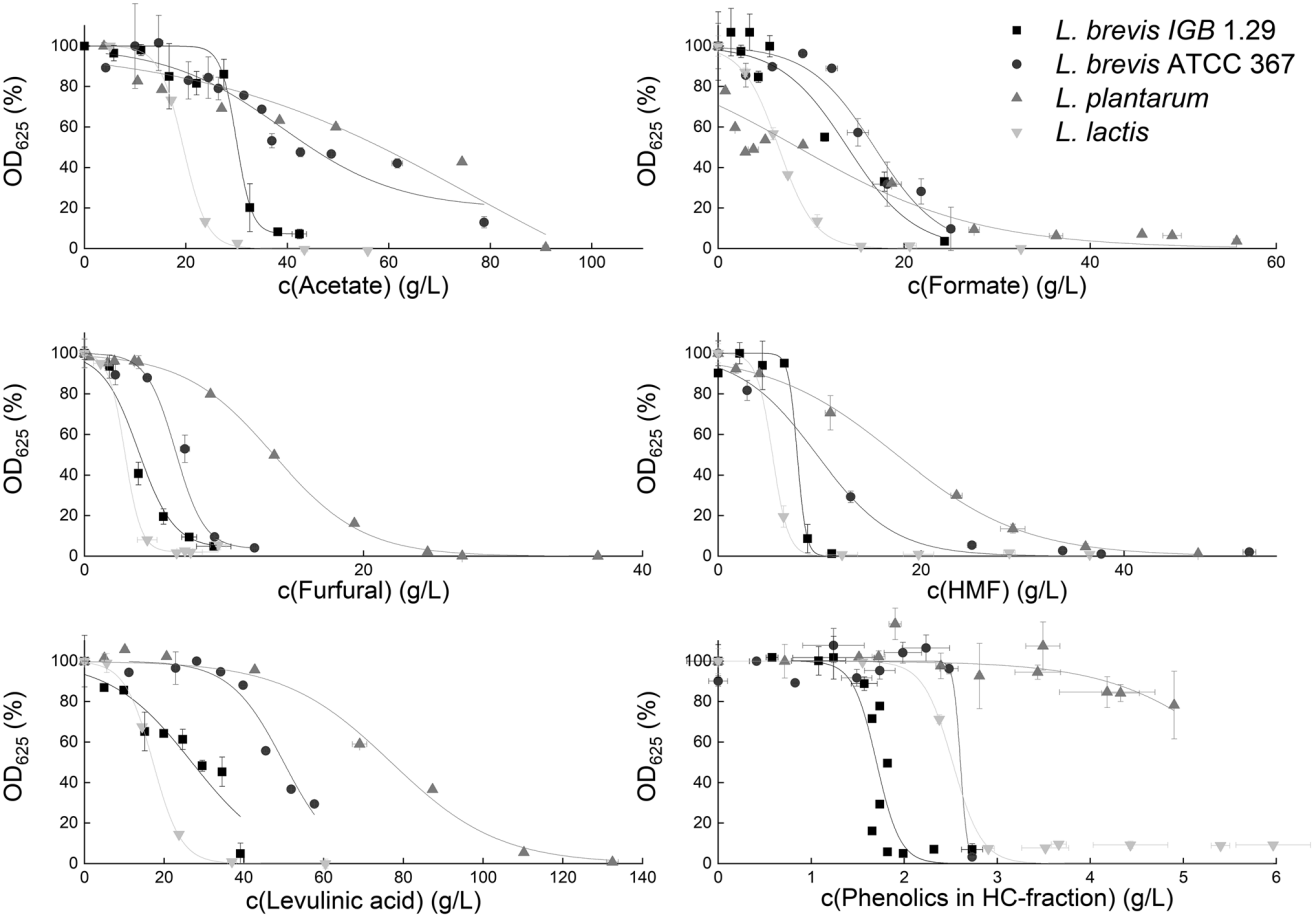
Effects of lignocellulose degradation products on cell growth *of L. brevis* IGB 1.29, *L. brevis* ATCC 367, *L. plantarum* NCIMB 8826 and *L. lactis* IO-1 JCM 7638 visualized as dose-response curves

The evaluation of the microorganisms’ tolerance towards lignocellulose degradation products with respect to cell growth was carried out at different concentrations of acetate, formate, furfural, HMF, levulinic acid or HC-fraction compounds. Non-inhibited growth is set to 100%. Results are shown in the form of dose-response curves in Fig. 2.

*L. brevis* IGB 1.29 was highly tolerant towards acetate. The IC_10_ of acetate was determined as 25.5 g/L. Compared to the other strains the data generated revealed a decreasing tolerance in the order *L. brevis* IGB 1.29 (25.5 g/L), *L. brevis* ATCC 367 (19.2 g/L), *L. lactis* (10.2 g/L) and *L. plantarum* (9.0 g/L). Formate affected the growth of the microorganisms already at lower concentrations. A 10% inhibition was detected for *L. brevis* IGB 1.29 at a concentration of 10.3 g/L, whereas the concentration for IC_10_ of *L. plantarum* already was at 0.2 g/L. In contrast, the IC_10_ of 6.9 g/L determined for *L. plantarum* in the presence of furfural was higher than the values obtained for the other bacteria. A higher tolerance could also be shown in the case of levulinic acid and HMF with IC_10_ values of 42 g/L and 8.0 g/L, respectively.

As many different phenolic compounds can be present in the liquid hemicellulose fraction after pretreatment [50], growth experiments were carried out with a dilution series of this fraction to adjust different total phenol concentration. It has to be taken into account that the fraction contained other inhibitory substances as well, but each below inhibitory concentrations according to our investigations (see above). However, synergistic effects between the toxic compounds may exist and the IC_10_ values can therefore not exclusively be attributed to phenolics alone. The application of the complex hydrolysate fraction thus enables a more realistic general assessment of the lignocellulosic hydrolysate rather than using one specific phenolic compound.

The data of cultures containing hemicellulose fraction revealed that *L. plantarum*, compared to the other tested microorganisms, showed a high tolerance and exhibits an IC_10_ at 3.7 g/L. Since *L. plantarum* showed robust growth in presence of HMF, furfural and phenols, it can be stated that the strain shows a high tolerance towards aromatic compounds in general. Drawback of this strain is the low IC_10_ value for formate being significantly below the other microorganisms’ values. Thus, formate would need to be removed to apply *L. plantarum* for the conversion of monosaccharides into lactic acid in hemicellulose fractions. *L. brevis* ATCC 367 showed 10% inhibition of cell growth at a phenol concentration of 1.44 g/L. In general, *L. lactis* showed the highest sensitivity towards the presence of lignocellulose degradation products. In order to increase the tolerance, there is the opportunity to adapt microorganisms to toxic substances in repeating subcultivations or in continuous fermentation as shown for *P. stipites, S. cerevisiae*, *Z. mobilis* strain 8b and *Scheffersomyces stipitis* [48, 49, 51, 52].

The inhibitory concentrations determined for the lactic acid producers presented in this study are in a comparable range to other authors’ results for *Pseudomonas taiwanensis* VLB120, *Candida tropicalis*, and different *Lactobacillus* species [53–55]. An overview of a selected literature using *L. brevis*, *L. lactis* and *L. plantarum* and investigating glycose, xylose or LC-hydrolysates and shown sensitivity to LC-degradation compounds is shown in Table 2.

**Table 2:** Overview of selected literature using *L. brevis*, *L. plantarum* and *L. lactis* investigating growth on lignocellulose hydrolysates and/or measuring sensitivity against LC-degradation products

	Ssp.	C-source	Sensitivity against LC-degradation products	Yield (g/g)	Product. [g/(L h)]	Literature
*L. brevis*	ATCC 367	Glucose, xylose^a^	–	0.52–0.70	0.36–0.58	[27, 29, 56]
	S3F4	Glucose, xylose^a^	< 20 mM furfural	–	0.68	[36]
	ATCC 14869	Glucose, xylose^a^	–	1.01	–	[35]
*L. lactis*	DSM 20481	Glucose	4% growth with 4 g/L HMF; 7% growth with 5 g/L furfural, 122% growth with 5 g/L acetate; 74% growth with 10 g/L levulinic acid^b^	–	–	[18]
	IO-1 JCM 7638	Glucose, xylose	–	–	–	[26]
		Xylose	–	–	0.68	[57]
*L. plantarum*	Isolated strain	LC-hydrolysates	Growth possible with 8 g/L furfural, 6 g/L HMF +	–	Up to 1.9	[17]

^a^Parallel consumption shown

^b^Glycolic acid, formic acid, acetosyringgone, syringaldehyde, vanillin, benzaldehyde, coumaric acid, ferulic acid were also tested

– not shown

+ there were also tested: vanillin and syringaldehyde

It should be noticed that in the presented work, the phenol experiments were done with a complex mixture. Synergistic effects of these various compounds might have led to decreased tolerance of the lactic acid bacteria presented in this work compared to the single model substance vanillin. However, the investigations performed within our study provided increased significance with regard to a prospective application of lignocellulose-derived fractions.

The reported growth data indicate that the investigated lactic acid bacteria are much more tolerant towards lignocellulose degradation products compared to several other common bacteria and yeasts as discussed above. The determined threshold values will enable a targeted concentration-oriented adjustment of the inhibitory compounds by adapting pretreatment parameters or by targeted purification of the hydrolysate.

### Influence of Low pH

Microbial growth of the *L. brevis* IGB 1.29 has been investigated at different initial pH values to determine if cultivation at pH < 6 is feasible. So far, only inconsistent results for cell growth of *L. brevis* at various culture conditions with regulated or unregulated pH have been published [4, 5, 11, 12]. The pH optimum of 5–7 (set to 100% growth) was identified by microbial cultivation at different starting pH-values. At pH 4 and without inhibitors, the microbial growth inhibition equaled 19% compared to growth at pH 6. Lactic acid production has not been affected at pH 4 for the selected culture conditions. At pH 3 no growth was detected (data not shown). These results show that lowering the pH from pH 6 to pH 4 is feasible for the lactic acid fermentation process for *L. brevis* IGB 1.29. According to the Henderson-Hasselbalch equation lowering the pH from pH 6 to pH 4 would result in an equilibrium shift to the protonated form of lactic acid from 0.7% at pH 6 to 41.5% at pH 4. Hence, in the obligate step of acidification during recovery of the protonated product after fermentation, the amount of added acid could be reduced by 40%, as well as the by-product formation.

Lactic acid fermentation from lignocellulose hydrolysates at low pH does not only influence cell growth and the amount of protonated lactic acid in solution but also the chemical state of the lignocellulose degradation products. The influence of pH 4 on the toxicity of the lignocellulose degradation products acetate, formate, levulinic acid, HMF, furfural and hemicellulose faction with phenolic compounds was tested for *L. brevis* IGB 1.29 and compared to the results at pH 6 (see Fig. 2). Results are shown in Table 3.

**Table 3:** Summary of the inhibitory concentrations of the lignocellulose degradation products acetate, formate, furfural, HMF, levulinic acid and phenolic compounds leading to 10% (IC_10_), 50% (IC_50_) and 90% (IC_90_) inhibited cell growth of *L. brevis* IGB 1.29 at different pH values (pH 4 and pH 6)

	pH	IC_10_ (g/L)	IC_50_ (g/L)	IC_90_ (g/L)
Acetate	4	6.4	9.8	13.4
	6	25.5	30.2	37.5
Formate	4	0.3	1.1	2.2
	6	4.8	12.4	21.1
Furfural	4	1.5	2.7	4.0
	6	1.2	3.7	7.3
HMF	4	5.8	8.5	10.6
	6	6.6	7.9	9.2
Levulinic acid	4	7.9	9.4	11.1
	6	9.2	25.7	38.0
HC fraction with phenols	4	0.8	0.9	1.0
	6	1.4	1.7	2.1

The curves are shown for pH 6 in Fig. 2

For acetate, the IC_10_ value at pH 6 (25.5 g/L) was about four times higher than at pH 4 (5.3 g/L). Even more obvious was the influence of the pH value for formate. At pH 6, 4.8 g/L formate led to 10% inhibition of cell growth. This is a 14 times higher tolerated concentration than at pH 4 (0.3 g/L). Concerning furfural and HMF, the influence of the pH value was less significant. The IC_10_ of furfural has been increased at pH 4 from 1.2 g/L (pH 6) to 1.5 g/L. However the IC_90_ of 4.0 g/L (pH 4) was increased to 7.3 g/L at pH 6. For HMF the IC_10_ of 6.6 g/L at pH 6 was lowered to 5.8 g/L at pH 4. Concerning levulinic acid, there has only been a slight influence of the pH value on the IC_10_ value (7.9 g/L at pH 4 and 9.2 g/L at pH 6). However, the IC_90_ showed a much lower concentrations at pH 4 (11.1 g/L) compared to pH 6 (38.0 g/L). The influence of phenolic compounds was twice as high at pH 4 than at pH 6. The IC_10_ at pH 4 was already reached at a concentration of 0.8 g/L phenolic compounds compared to 1.4 g/L at pH 6. It was similar for the IC_90_ value at 1 g/L for pH 4 and at 2 g/L for pH 6.

Consequently, pH had a strong influence on the toxicity of the organic acids acetate, levulinic acid and formate which is due to their p*K*_a_-values and of the hemicellulose fraction (with phenolic compounds). The toxicity of the organic acids depends on the diffusion of the protonated acids into the cell, where they cause a decrease of pH affecting intracellular processes. Compared to the other organic acids acetic and levulinic acid, formate revealed the largest influence regarding cell growth. At pH 4 the IC_10_, IC_50_ and IC_90_- values have been 90% lower than at pH 6. Compared to the other organic acids, formate has the lowest p*K*_a_-value (3.75) but also the lowest molecular weight (46.03 g/mol) so it can easily diffuse into the cell. Applying acetate at pH 4 the IC_10_, IC_50_ and IC_90_ was decreased by 70% compared to pH 6. For levulinic acid the low pH affected only IC_50_ and IC_90_ significantly by a reduction of 65–70% compared to pH 6. The p*K*_a_-values of acetate (p*K*_a_ = 4.75) and levulinic acid (p*K*_a_ = 4.66) are very similar. The stronger influence of acetate might again depend on the molecular weight which is 60.05 g/mol for acetate in respect to 116.11 g/mol for levulinic acid. Supporting these statements, Huang et al. had described the organic acids as main inhibitors for yeasts especially at low pH, while the hemicellulose fraction (with phenolic compounds) had a minor influence [58].

The toxicity of phenolic compounds has been shown to be increased by low pH. At pH 4 the IC_10_, IC_50_ and IC_90_ values were 50% lower than compared to pH 6. The decomposition of lignin leads to phenolic acids, alcohols, aldehydes or ketones, depending on the side groups of the lignin [59, 60]. The hydroxyl groups of phenolic aldehydes and ketones have the lowest p*K*_a_-value of 7.3–8.2. The p*K*_a_-value of the hydroxyl group of phenolic acids is 9–11. The p*K*_a_-value of the acid group is between 3.4 and 4.6 [60]. This means that already at pH 6 all hydroxyl groups of the phenolic compounds are protonated. Mussatto et al. stated that an additional decrease in pH only influences the protonation of the acid group of phenolic acids [61], which is 50% at pH 4 and 0.1% at pH 6 leading to higher toxicity at pH 4. It has to be taken into account that the fermentations were carried out with a dilution series of the hemicellulose fraction of the organosolv process to adjust different phenol concentrations. This is why the fraction contained other inhibitory substances also being affected by pH.

Huang et al*.* considered the inhibiting effect of a total hydrolysate on *S. cerevisiae* at different pH values (pH 4.5–9) without differentiation between the potential inhibitors. Within these investigations, the influence of the pH value on the hydrolysate toxicity could be demonstrated [58]. The data presented here confirm the influence of pH on the toxicity for each single lignocellulose degradation product. On the basis of the single compound concentrations in the hydrolysates, the inhibition of *L. brevis* IGB 1.29 being cultivated at pH 4 can be predicted.

The wheat straw-derived hemicellulose fraction in this work led to significant inhibition of *L. brevis* ATCC 367 at pH 4 in particular because of 5.1 g/L phenolic compounds. Taking the results of this work into account, and not considering potential synergistic effects of different lignocellulose degradation products, this concentration has to be reduced to 1.4 g/L for a successful cultivation at pH 4. The concentrations of all other lignocellulose degradation products are not inhibiting.

## Conclusion

*Lactobacillus brevis* IGB 1.29 has been isolated from compost samples. The strain was characterized and compared to *L. brevis* ATCC 367, *L. plantarum* and *L. lactis* with regard to xylose and glucose metabolism and tolerance towards several common toxic compounds. *L. brevis* IGB 1.29 is able to co-metabolise glucose and xylose with comparably high xylose consumption rates. The tolerances towards each single lignocellulose degradation product, acetate, formate, levulinic acid, HMF, furfural and phenolic compounds was determined and exceeds in general the IC values reported for other tested lactic acid bacteria and published results. Inhibitory concentrations were also measured at pH 4, which is the lowest pH at which cultivation of *L. brevis* IGB 1.29 is feasible. Important thresholds for the concentrations of lignocellulose degradation products have been determined to select suitable lignocellulosic raw materials and pretreatment methods with adapted concentrations of toxic compounds.
